# Mucosal Vaccination via the Respiratory Tract

**DOI:** 10.3390/pharmaceutics11080375

**Published:** 2019-08-01

**Authors:** Marie Hellfritzsch, Regina Scherließ

**Affiliations:** Department of Pharmaceutics and Biopharmaceutics, Kiel University, Grasweg 9a, 24118 Kiel, Germany

**Keywords:** mucosal immune system, dendritic cells, formulation, adjuvants, nanoparticles, delivery device

## Abstract

Vaccine delivery via mucosal surfaces is an interesting alternative to parenteral vaccine administration, as it avoids the use of a needle and syringe. Mucosal vaccine administration also targets the mucosal immune system, which is the largest lymphoid tissue in the human body. The mucosal immune response involves systemic, antigen-specific humoral and cellular immune response in addition to a local response which is characterised by a predominantly cytotoxic T cell response in combination with secreted IgA. This antibody facilitates pathogen recognition and deletion prior to entrance into the body. Hence, administration via the respiratory mucosa can be favoured for all pathogens which use the respiratory tract as entry to the body, such as influenza and for all diseases directly affecting the respiratory tract such as pneumonia. Additionally, the different mucosal tissues of the human body are interconnected via the so-called “common mucosal immune system”, which allows induction of an antigen-specific immune response in distant mucosal sites. Finally, mucosal administration is also interesting in the area of therapeutic vaccination, in which a predominant cellular immune response is required, as this can efficiently be induced by this route of delivery. The review gives an introduction to respiratory vaccination, formulation approaches and application strategies.

## 1. Introduction

Active immunisation, which can achieve a lifelong immunity, is one chance to prevent infectious diseases. It has proven to be one of the most cost-effective public health interventions through the years and the number of immunised children is continuously increasing. Thus, the World Health Organisation (WHO) specifies that approximately two to three million lives could be saved every year because of immunisation and the highest number of vaccinated children was reported in 2017: 116.2 million [1].

One of the most recent examples of how vaccinations are important to prevent diseases is the measles vaccination. Measles are a viral illness and one of the most infectious diseases, especially in young children. Since the 1960s, where the first measles vaccine was used, the number of cases worldwide decreased from almost 4.5 million in 1980 to 90,000 in 2016 because of different types of measles vaccines, regardless of whether mono or combination preparations were used [1,2]. But in 2018, almost 83,000 cases were reported just in Europe again and, according to WHO, that is three times higher than in 2017 and 15 times higher than in 2016 [3]. Increasing outbreaks show that hard-won gains are easily lost. Even in countries with well-established health and national immunisation systems, political and economic changes can have an influence on vaccination status without constant attention [1]. Two of the main goals of the Global Vaccine Action Plan of the WHO [1] are increasing vaccination rates again and treating other infectious diseases with vaccines. The focus is on the research and development of vaccines against diseases that are widespread worldwide, such as malaria and HIV as well as new technologies. While Mosquirix^®^, a vaccine against malaria, has received positive scientific feedback from the European Medicines Agency and is now being tested in studies in three African countries, the vaccine development for HIV is more challenging in a technological view, because of great variability and mutability. However, regarding HIV vaccines, multiple candidate vaccines are in clinical and pre-clinical evaluation.

As already mentioned, vaccines can be divided into mono vaccines and combination vaccines. Another possibility of subdivision is the division into the following four groups: live attenuated (e.g., measles, oral polio virus), inactivated (e.g., whole-cell pertussis, inactivated polio virus), subunit (e.g., hepatitis B, pneumococcal) and toxoid (e.g., tetanus toxoid, diphtheria toxoid). Live attenuated vaccines are produced from disease-causing pathogens and weakened under laboratory conditions. They can grow and cause no or very mild infection in vaccinated individuals. The immune response is almost as good as that of the original pathogen as all pathogen components are present. However, it is conceivable that this may go hand in hand with negative effects. Live attenuated vaccines have the rare potential to revert to the pathogenic form and cause diseases or adverse effects, immunosuppressed individuals may not be able to react to the antigen and they should also not be administered during pregnancy. In connection with pregnancy and childbirth, another problem may arise during vaccination: maternal antibodies [4]. Early in life, infants have an immature immune system and maternal antibodies are transferred from mother to child. These antibodies inhibit vaccination of the newborn and provide inadequate protection despite vaccination with different mechanism. In order to avoid inactivation and inadequate vaccination protection, different strategies are discussed, including maternal immunisation. It has the advantage that the maternal immune system will react well to vaccination and, thus, provides high levels of antibodies for the infant [4]. In addition, the vaccination of the mother would be possible through various methods of administration.

Because of a reliable systemic immune response and exact dosing possibilities, intramuscular administration is the most used method. Due to the fact that it is an invasive procedure, usage of a needle is always necessary. Hence, trained personnel for administration and a sterile dosage form are indispensable and undesired effects can occur as a result, e.g., accidental needle sticks. By far the biggest problem associated with injury to an infected needle is the transmission of infectious diseases, e.g., HIV, hepatitis, and the risk of sepsis [5].

In his review on needle-free immunisation, Mitragotri [5] shows various alternatives to intramuscular application (Table 1). Cutaneous and mucosal immunisations are possibilities of non-invasive administration. Particular emphasis should be placed on the mucosal immunisation via the respiratory tract, because pathogens mostly have their first contact with nasal and pulmonary mucosa when entering the human body.

This review takes a closer look at mucosal immunisation via the respiratory tract as an alternative to intramuscular application. Different aspects will be investigated, starting with the immune system of the respiratory tract and particle uptake through formulations to therapeutic vaccination.

## 2. The Immune System of the Respiratory Tract

To understand the function of the mucosal immune system, it is important to look at the structures of the upper (from the nasal and oral cavities to the throat) and lower (trachea and lung) respiratory tract. It should be noted that there are large differences between species and age groups. The immune system in the upper and lower respiratory tract can be structured in three parts (Table 2). As mentioned before the first parts are epithelial compartments with immunocompetent cells with four different cell types: alveolar macrophages, dendritic cells, M cells and intraepithelial lymphocytes. The second parts are the lymphoid structures of the nose and the bronchus: nose-associated lymphoid tissue (NALT), larynx-associated lymphoid tissue (LALT) and bronchus-associated lymphoid tissue (BALT). The third parts are lymph nodes that drain the respiratory system [6].

Pavot and coworkers [7] divide the mucosal immune system into inductive and effector sites. At the lamina propria, which is the effector site level, effector cells control foreign material and secretory antibodies are produced, especially IgA. The secretory IgA is very important in preventing infections, because it inhibits the adhesion of bacteria, viruses and other pathogens to epithelial cells. Immunoglobulin G is also produced and plays a role in neonatal immunity, because there is a passive delivery of IgG from mother to child. Intraepithelial T lymphocytes are concentrated in the surface epithelium; NK-like cells, macrophages and B and T cells are located in the sub-epithelial compartment; antigen presenting cells and dendritic cells are present in the mucosal lymphoid tissue. At the inductive site, which is the mucosa-associated lymphoid tissue (MALT), an immune response is initiated. The MALT is the biggest lymphoid tissue in the human body with 4/5 of all immune cells [8] and has three main functions:

1. Protect the mucosal surface against invasion by microbial pathogens;
2. Prevent internalisation of commensal bacteria or antigens as non-degraded proteins;
3. Induct tolerance against innocuous soluble substances.

### 2.1. Immune System in the Nose

As the respiratory tract and especially the nose as part of the upper respiratory tract is one of the main entry ports for pathogens, it is well equipped with immunocompetent cells. The lymphoid tissue of the nose (nose-associated lymphoid tissue, NALT) is located in the nasopharynx and the tonsils as part of the Waldeyer’s ring [6,9]. It is part of the common mucosal immune system, being characterised by circulation and homing of mature immune cells to the different mucosal sites [10], and can induce a local immune response in the respiratory tract as well as systemic immune reactions [11,13]. The NALT is characterised by a reduced number of ciliated and goblet cells compared to the normal respiratory epithelium present in the nose, numerous intraepithelial T and B lymphocytes and some macrophages [9]. It is known that in these areas, specialised epithelial cells allow intimate contact with lymphoid cells and antigens are taken up following this route to be presented to underlying dendritic cells [13]. Whereas NALT is located in confined structures, dendritic cells (DCs) as antigen presenting cells are also present throughout the complete epithelium. They might congregate immediately under epithelia, migrate into the epithelial layer and even extend dendrites into the lumen to capture antigens. Dendritic cells will then travel to the nearest draining lymph nodes, the cervical lymph nodes, to present the captured antigen to T cells [12]. Upon uptake the antigen is processed by a DC, which then will stimulate CD4-positive T cells to induce IgA-committed B-cell development in the lymphoid follicle. After maturation, these B cells migrate from NALT to the regional cervical lymph nodes. Finally, antigen-specific CD4-positive T cells and IgA-positive B cells migrate to effector sites (such as the nasal passage) through the thoracic duct and blood circulation [14].

### 2.2. Immune System in the Lung

In the lung, MALT structures (here named bronchial-associated mucosal tissue, BALT) can be found along the upper and lower airways, where they are mostly stretched out at the branching sites, where turbulent flow occurs which enhances the likelihood of antigenic particles impacting on these structures [9]. Within this lymphoid tissue the number of ciliated cells is reduced compared to normal respiratory epithelium, whereas numerous lymphocytes and macrophages are present. The frequency of immunocompetent cells varies between the upper bronchial airways, where a dense network of those cells can be found, and the lower respiratory tract with an increased number of alveolar macrophages compared to dendritic cells. Whereas macrophages play an important role in the first line defence of the innate immune system, dendritic cells are key players in adaptive immune responses [15]. Dendritic cells are found in the epithelial linings of the conducting airways, in the submucosa, within alveolar septal walls as well as on the surface of the alveoli [16]. From the cells in the conducting airways, about 2% of the total cell population are airway mucosa dendritic cells, whereas only 1% of the cell population in the lung parenchyma are dendritic cells. Nonetheless, there are indications that these cells are more efficient in antigen uptake than the DCs of the upper airways [15]. Due to the differences in distribution of the different immune cells throughout the lung, a targeted delivery to certain cells may be possible, which in turn can induce the desired immune response.

## 3. Advantages of Mucosal Immunisation

Inhalable pathogens enter the body or directly infect the host via the mucosal surface. For this purpose, it would be interesting to achieve an immune response at the mucosal surface. The problem is that intramuscular vaccination often creates a poor mucosal immune response [17]. Nasal and pulmonary delivery are the most effective routes to reach a local immune response besides a systemic immune response. That means mucosal immunisation produces antigen-specific IgA antibodies at the infection site as well as IgG for an additional systemic response and defence mechanism against the microorganism [18]. Furthermore cell-mediated responses can be stimulated, e.g., helper CD4-positive T cells and CD8-positive cytotoxic T lymphocytes [18].

The nasal mucosa is a very interesting route of drug delivery [19]. Partidos [20] mentioned in his review different reasons why the nose is an attractive route for immunisation:

- Easily accessible;
- Highly vascularised;
- After intranasal immunisation, both mucosal and systemic immune responses are induced;
- Immune response can be induced at distant mucosal sites owing to the dissemination of effector immune cells in the common mucosal immune system;
- Can be used for the immunisation of large population groups;
- Does not require needles and syringes, which are potential sources of infection.

Lu and Hickey [16] confirmed the reasons for nasal immunisation also for pulmonary immunisation and complement the arguments of Partidos:

- Rapidly immunisation of the population;
- Avoid the risk of transmitting hepatitis B, HIV and other blood-borne diseases;
- Highly responsive immune system.

Mucosal immunisation is a promising application technique, because of a rapid onset of action, activation of all the different arms of the immune system, fewer side effects and all the practical advantages [6,18,21,22].

## 4. Prerequisites for Mucosal Vaccination

For mucosal vaccination, there are some specific requirements, which differ from parenteral vaccination. Most importantly, the antigen needs to be presented in a particulate form in order to provoke local and systemic immune response. Further, an adjuvant with mucosal effectivity is needed. Finally, the formulation needs to be capable for respiratory delivery via a suitable device, deposition at the target site and interaction with the immune cells.

### 4.1. Particle Uptake in Immune Cells

To induce an immune response in the respiratory tract, the antigen has to be taken up by antigen-presenting cells (APCs) in a particulate form [23]. This is easy if the antigen itself is a particle similar to an attenuated or inactivated pathogen, but it is more complicated for subunit vaccines, where the antigen needs to be formulated with a particulate vaccine carrier. Dendritic cells are efficient stimulators for B and T cells [24]. DCs are able to take up microbes and particles by phagocytosis, further they are able to form large pinocytotic vesicles incorporating extracellular fluid and solutes with this taking up soluble antigens by macropinocytosis. They are also equipped for targeted uptake following receptor-mediated endocytosis via C-type lectin receptors or mannose receptors [23]. Upon uptake, DCs are able to form large amounts of peptide-MHC II complexes which are then shown on the cellular surface instead of degrading protein antigens in lysosomes.

### 4.2. Particle Size

De Temmerman and coworkers [23] describe the targeting of particulate antigens to dendritic cells, which are the most potent and versatile antigen presenting cells. The corresponding particle size ranges from nanometres to the lower micrometre range. For particulate uptake in DCs, it can be shown that there is a certain size-dependent efficacy in terms of immune response, as smaller particles of about 200 nm provoke a higher immune response than larger particles of 700 nm [25]. Own studies found a particle size of 200–300 nm to be optimal for dendritic cell uptake [26]. Smaller particles in a virus-like size (20–200 nm) are taken up by endocytosis and rather provoke a cellular immune response, whereas very small nanoparticles are not processed locally, but are directly drained to the lymph nodes similar to soluble antigens [23]. This results in local tolerance and a predominantly systemic immune response, if at all [27]. Larger particle (>0.5 µm) are absorbed by phagocytosis or micropinocytosis and lead to a bacteria-like humoral response [28].

Nonetheless, nanoparticles, as discussed in more detail in Section 9.3, may only carry a small antigen load. This can be increased largely with microparticulate carriers, which are not very well taken up by DCs, but can be taken up by M cells in the MALT. Tafaghodi and coworkers [29] report on the size analysis of microspheres and how size influences the uptake by M cells. Particles up to 10 µm are taken up. Furthermore, the sizes are subdivided with regard to their immune response. While particles smaller than 5 µm stimulate the mucosal and systemic immune responses, particles in the range of 5–10 µm stimulate only the mucosal immune system. Some studies implicate, that particles larger than 5 µm can be taken up by M cells, but will remain in these cells rather than being transferred to underlying APCs [30].

Furthermore, Fifis and coworkers [31] and Singh and coworkers [32] published papers on size-dependent immunogenicity and opportunities and challenges of pulmonary route for vaccination. They report on size restrictions for uptake in macrophages of particles in the micrometre range starting from 0.5 µm. Alveolar macrophages are reported to be especially efficient in the uptake of particles between 3 µm and 6 µm [33]. Therefore, for DC-targeted uptake in the respiratory tract the particle size should be lower than 3 µm, best lower than 0.5 µm.

### 4.3. Particle Shape

Apart from size, the shape of the particles also plays a role for cellular uptake [34]. When particles of different aspect ratios are compared, it was shown that elongated particles are taken up better than spherical particles or cylindrical particles and that disc-shaped particles are taken up to the least extent [35]. Aspect ratio will not only determine the ratio of volume to surface, but can also determine particle orientation and recognition [36]. For macrophages it could be shown that the particle extension at the point of cell attachment is more important for uptake than the total size [35]. Shape could also play an important role in intracellular particle transport. Here, a shape not correlating with intracellular actin- or microtube-dependent transporting could prevent further particle processing. In a study from Liquidia, the immunological response on antigens being formulated in differently shaped PRINT particles was looked at and they found, that the antibody titre was highest from earthworm-like particles compared to cylindrical, hexnut formed or cubic particles [37].

### 4.4. Charge and Functionalisation

Further characteristics such as charge and functionalisation of the particles also play a role in the efficacy of uptake [38]. Generally, positively charged particles can better interact with negatively charged cell membranes than negatively charged particles, with this increasing contact time and mediating uptake [39]. This effect can also result in a higher immune response of cationic particles in vivo [40]. Whether cationic particles exceed uncharged particles is discussed controversially and may also depend on hydrophobicity of the particle [41]. Particle functionalisation comprises PEGylation which may increase stability of the particulate system and minimise aggregation [42], and also comprises attachment of receptor ligands for targeted cell interaction and uptake [43] such as lectins, toll-like receptor (TLR) ligands and other pathogen-associated molecular patterns (PAMPs) [44], which may also serve as adjuvants because of their immunostimulatory effects.

In areas where both DCs and macrophages are present such as the epithelium of the respiratory tract, uptake into macrophages should be avoided as these cells are not presenting antigen to subsequent immune cells [15], but predominantly clear the respiratory tract from particulate waste. It is reported that the phagocytic activity of macrophages is generally higher than of DCs, but uptake in DCs can be enhanced if the particle carries a positive charge [45].

## 5. History of Respiratory Vaccination

First reports on respiratory vaccination (called “variolation”) reach back to ancient China and India as early as 100 BC, where a treatment with powdered scabs from patients infected with smallpox for the protection of other people is reported [46]. The scabs of patients’ smallpox lesions of the skin were collected and dried with this attenuating the virus and ground to a powder. The powder was then administered to the nose of non-infected patients by a small blow pipe or it was air-dispersed for inhalation. This treatment was not harmless and bore a risk for a deadly smallpox infection of 0.5–2%, but compared to a mortality of 20–30%, when the disease was obtained through natural infection, this was a great success. For long, this application remained the only reported respiratory vaccination and it was in use in India until the 1970s.

In the last century, major trials were performed in the former Soviet Union where several thousand humans were successfully aerosol vaccinated over a period of many years with live-attenuated strains such as dry anthrax spores [16,47]. The first major clinical trial was probably performed by Albert Sabin and colleagues in Mexico in the 1980s when around 4 million children were vaccinated by a nebulised attenuated Edmonston Zagreb measles vaccine. This trial and the following trials showed that the immune response in children following the aerosol route of delivery was superior to injection [48]. They used a custom-made device for aerosolization, which made use of the commonly available product for injection, which was reconstituted and had to be kept on ice during nebulisation in order to keep it stable.

These examples prove the respiratory tract to be highly interesting as route of vaccine administration and many more pre-clinical and clinical trials are currently being undertaken to strengthen the scientific basis for respiratory vaccination as well as to develop vaccine products for respiratory vaccination for disease prevention and in the therapeutic field.

## 6. Formulations for Respiratory Vaccination: General Considerations

Unlike parenteral vaccine formulations, which have to be injectable liquids, formulations for the respiratory tract comprise solutions or suspensions administered by nasal sprays, pressurised nasal sprays/pressurised metered dose inhalers or nebulisers as well as dry powder formulations which can be administered by passive or active dry powder dispensers. However, for all formulations, whether liquid or solid, it is important that the antigens are presented in a particulate form to induce a local and systemic immune response. Various factors influencing particle properties are discussed in Section 4.

Liquid preparations face a high risk of instability over storage due to the high molecular mobility and with this, increased likelihood of chemical reactions and physical instability [49]. This is the reason why many liquid vaccine preparations need to be stored and transported under refrigeration. For a liquid antigen preparation, stability can be enhanced by a selection of pH buffering salts and often amino acids are also used for stabilisation [50,51]. The aim is to ensure and maintain optimal antigen hydration without physical instability or chemical degradation. Hence, osmolarity may play a critical role. For the respiratory mucosa, the osmolarity and pH of the administered liquid (solution or suspension) are also important parameters, as preparations, which deviate largely from physiological conditions, may cause irritancy. In addition, it has been shown that buffer ionic strength may influence the uptake of nanoparticles into M cells [52].

Stability, especially thermal stability, can be increased largely if the antigen can be stabilised and dried. Liquid preparations are often freeze dried to enhance storage stability, with this minimising molecular mobility and, hence, risk of intermolecular reactions. If they shall be administered in liquid form, they need to be re-dispersed in buffer directly prior to administration. With this, the antigen needs to be stable in liquid and has to be stabilised during freezing (cryoprotection) and the subsequent drying step (lyoprotection) [53]. During freezing the molecule needs to be protected from harmful effects of the forming ice crystals and a shift in pH, which may easily occur due to the formation of saturated solutions differing in salt composition from the original buffer during freezing [54]. Afterwards, the molecule needs to be stabilised from dehydration during the removal of water. This can be achieved by an exchange of water with other hydrophilic molecules which may replace it as hydrogen bond forming partner. Another possibility is the formation of a sugar glass matrix, which has been shown to stabilise vaccine preparations [55]. This principle can also be used in other drying techniques such as spray-freeze drying or spray drying. Formulations, which shall be administered as dry powder, face the same problem as intermediate formulations in the dried state: the antigen and its carrier system need to be stabilised during drying. Amorij and coworkers [54] describe different possibilities to stabilise vaccines. As protein formulations are more stable in a solid state than in a liquid state, they discuss different sugars, which can stabilise proteins during spray and freeze drying (Table 3). The described mechanisms and stabilisers are not only used in influenza vaccines, but also in numerous other formulations in literature [56,57]. Furthermore, the dried formulation needs to have a particle size allowing deposition in the targeted area of the respiratory tract, should have good dispersion characteristics and low agglomeration and adhesive tendencies to allow powder handling, packaging and efficient release from the device. Particle size can be controlled by the parameters of the drying procedure. Here, processes resulting in a dispersible dry powder in one step (such as spray drying) are favoured to freeze drying, where the freeze-dried cake might need to undergo a milling step to obtain the desired particle size [58,59]. Cohesive and adhesive behaviour are, in part, determined by particle size: the larger the particles, the better their flow characteristics and the lower their agglomeration. Powder characteristics can further be controlled by the use of dispersion modifiers, which either cover the surface of the microparticles resulting in reduced hygroscopicity and surface energy [60,61,62] or which form separate particles in the dry powder [63] increasing the dispersion capability.

Its particulate carrier may also achieve stability of the antigen. Drying nanoparticles without further bulking excipients normally leads to highly aggregated particles of undefined size, which are difficult to re-disperse and have a very low yield. Therefore, further excipients can be added which serve as a matrix, embedding and stabilising the individual nanoparticles in a Nano-in-Microparticle (NiM) formulation and increasing re-dispersibility upon matrix dissolution [64]. The matrix component should consist of a material which is capable of quickly releasing the particulate vaccine carrier upon dispersion in media or deposition on the respiratory mucosa. Normally, this is secured by the use of water-soluble carbohydrate matrices, which dissolve in the aqueous mucus.

## 7. Adjuvants for Respiratory Vaccines

Antigens for mucosal vaccination often require an adjuvant to achieve both systemic and local immune response. Adjuvants are substances that have to be administered together with the antigen in the formulation to obtain the desired immune response [65]. This is of special need when the antigen does not bear a strong immunogenicity such as for subunit vaccines and purified antigens, especially for mucosal delivery routes. Here, the targeted epithelium, which naturally is in contact with many potential antigens, need a strong immunostimulating signal in order not to induce tolerance [26,66].

Further, adjuvants can be used to guide the immune response as they may determine whether a predominant Th1- or Th2-mediated immune response or an immune response based on cytotoxic T lymphocytes (CTL) is developed [27]. This is of special interest when a CD8-positive CTL-specific immune reaction is needed such as it is the case for therapeutic vaccines [67,68].

Chadwick and coworkers [69] present various mucosal adjuvants in their review of nanotechnologies, whereas only those listed in Table 4 can be used in the respiratory tract, and Lu and coworkers [16] talk about different mechanisms of action.

To be feasible as mucosal adjuvant, the substance should enhance immunogenicity of the co-administered antigen but should not have intrinsic immunoactivity or toxicity. It has to be noted, that enterotoxins such as the heat-labile toxin from *E. coli* or the cholera toxin, which have proven to be effective mucosal adjuvants, may not be used in the nose due to the possible neurotoxic effects [83]. Non-toxic mutants or derivatives such as MPL are a feasible alternative, but the efficiency for subunit vaccine preparations may be questionable [84].

## 8. Therapeutic Vaccination

Active immunisation utilises vaccination to prevent infectious diseases. Therapeutic vaccination provides a new tool for the treatment of cancer, autoimmune diseases or persistent infections, but it is much more challenging [85]. Several mechanisms are described for developing or influencing these diseases, e.g., genetic factors, exogenous factors and a dysregulated function of the immune system [86]. Cancer cells are characterised by the fact that they are genetically altered and have lost normal cellular regulatory processes. As a result, they tend to express different surface markers which could be recognised by immune cells followed by a presentation on MHC I complexes. These complexes can be recognised by CD8-positive T cells. In cancer patients, the immune system does not react in the right way. DCs and T cells may treat cancer antigens more as body’s own components than as foreign antigens. This leads to a T regulatory cell response rather than an effector response; T cells cannot infiltrate the tumour and factors in the microenvironment of the tumour suppress effector cells [87]. Here, therapeutic vaccines may help teach the immune system to regain its full function by (repeated) administration of a tumour-specific antigen along with immune-stimulating adjuvants and typically a tumour-suppressive chemotherapy [88]. As mentioned before, the therapy is usually not successful with a single administration, but typically is needed as a routine therapy. Further, a monotherapy by therapeutic vaccination is often not possible, because of the tumour-induced immunosuppression in the surrounding tissue and comedication by chemotherapy has to be administered [89].

Currently, there are many different approaches to therapeutic vaccination against cancer. Basically, a distinction must be made between three different approaches: cell vaccines (tumour or immune cell), protein/peptide vaccines and genetic (DNA, RNA, viral-based vectors) vaccines [88].

Tumour cell vaccines use, as the name suggests, tumour cells for vaccination. A differentiation has to be made between the use of the patient’s own tumour cells, which are removed, specially treated and re-applied (autologous tumour cell vaccines) and a whole tumour cell vaccine, which typically contains two to three typical cell lines (allogeneic tumour cell vaccines). In addition to tumour cell vaccines, there are also immune cell vaccines. Autologous DCs of the patients are loaded with tumour-associated antigens and the mature DCs are re-administered to the patients together with an adjuvant. Individualised tumour therapy sounds like a promising approach, but the availability of patient samples and the complex manufacturing process greatly limit its use [88].

Protein/peptide vaccines are recombinant vaccines containing peptides from defined tumour-associated antigens. They are then administered together with an adjuvant. Even if they are cheaper than individualised vaccines, they have a decisive disadvantage as they only target one or a few epitopes of the tumour-associated antigen and the one tumour type does not always have to be the same. An exception is the Stimuvax^®^, which contains CD4-related and CD8-related epitopes [88].

Genetic vaccines are another strategy to deliver antigen or fragments of antigens. After the administration of genetic vaccines, DCs that infiltrate the tissue during an inflammatory reaction are transfected, which leads to a direct antigen production and subsequent presentation. A distinction is made between DNA, RNA and viral-based vector vaccines. A big advantage is the easy administration of DNA or RNA encoding for different antigens and a complex activation of the immune system [88].

What all approaches have in common is that a rational vaccine design is needed to achieve a concentrated antigen delivery to DCs and effective DC activation leading to the induction of CD4-positive and CD8-positive T cell responses [85]. For this reason, a mucosal vaccination is to be preferred, since here a high cytotoxic effect and a good cellular immune response can be achieved.

## 9. Respiratory Vaccine Formulations

### 9.1. Nasal Vaccine Formulations

Primary antigen carriers are usually too small to be delivered directly to the nasal cavity, they would mostly get inhaled to the lung, hence, they need to be processed further to a formulation which can be deposited securely in the nose. Nanoparticles in suspension would be delivered within larger spray droplets. Spray droplet size will mainly be defined by the spray nozzle of the device as well as by further parameters like viscosity and surface tension of the dispersion medium. For nasal spray products, FDA guidelines require most of the spray droplets to be larger than 10 μm to ensure nasal deposition without a major postnasal fraction which would get inhaled to the lung [90]. For nasal dry powders, Hickey and coworkers [46] propose to use particles larger than 50 μm to ensure predominant nasal deposition. Dry nanoparticles tend to form uncontrolled agglomerates due to the large surface area, hence, they would be formulated in NiM particles. If dry powder Nano-in-Microparticle formulation, where the vaccine carrier is immobilised in a larger matrix particle, is directly administered to the nose, particles may cause physical irritancy depending on their size and concentration. Furthermore, all water-soluble components start dissolving in the nasal mucus. This may result in a concentrated solution of high osmolarity, which can also cause irritancy and increased ingression of water to dilute the substance causing a running nose. Finally, the nose is a highly sensitive organ for olfaction. Therefore, formulation smell is an important factor for patient compliance as well as taste, because all formulations will be cleared to the pharynx and will also be tasteable on the tongue. In order to increase nasal retention time and with this the time for interaction between the formulation and the nasal mucosa to allow uptake of particulate antigen preparations, mucoadhesive substances (hydrophilic polymers such as chitosan, hydroxypropyl methylcellulose or carbomer) can be used. Apart from the variability in formulations, another advantage of nasal administration is the difference in microbiological requirements as a nasal formulation does not need to be sterile. Further additives may comprise preservatives, which are mandatory for liquid multidose devices to ensure microbiological stability, and adjuvanting substances. Preservatives in nasal formulations are under controversial discussion especially in chronic use, as they may have an effect on ciliary function [91,92]. The choice of an effective and non-toxic adjuvant for nasal vaccination is a challenging task. Especially, it must be tested whether vaccine components can enter the central nervous system (CNS) and cause safety problems. It has been shown by molecular imaging for a botulism vaccine in monkeys that the antigen did not enter the CNS upon nasal administration [93]. The reports on Bell’s palsy following a nasal administration of an influenza vaccine adjuvanted with the heat labile *E. coli* enterotoxin (LT) are allocated to translocation of the adjuvant component to the CNS, which led to withdrawal of the vaccine from the market [94].

Fluenz Tetra^TM^ [95] is the first marketed respiratory vaccine. It is licensed in the EU for children (2–18 years) and FluMist Quadrivalent^®^ in the USA and Canada for children and adolescents (2–49 years) for the active immunisation against influenza disease. They are tetravalent vaccines with four influenza virus strains, which are cold-adapted, temperature-sensitive and attenuated. The selection of the virus strains is based on the annual recommendations of the World Health Organisation (WHO). These live virus particles are suspended in a buffered solution containing sucrose, gelatin and amino acids to increase stability. For application, a nasal spray syringe with 0.2 mL of the formulation is used, wherein 0.1 mL is administered into each nostril. Live attenuated influenza vaccines have to mildly infect and replicate in mucosal cells in order to protect. Because of presenting viral proteins in their native form, the immune response is similar to those by natural influenza infection. This results in a higher efficacy compared to inactivated vaccines in children [96] and also comparable protection for adults.

### 9.2. Pulmonary Vaccine Formulations

Similar to nasal formulations, the primary antigen carrier formulation needs to be processed further to obtain a product which is capable for efficient delivery to the lungs. This can be performed either as a liquid, which can be nebulised or delivered by a pressurised metered dose inhaler (pMDI) or as a dry powder formulation. Comparable to nasal formulations, aerodynamic behaviour and particle size of the administered formulation play an important role for the location of deposition. In general, an aerodynamic particle size between 0.5 µm and 5 µm is believed to be optimal for pulmonary delivery. Larger particles will already be deposited in the oropharynx, whereas smaller particles might get exhaled [97]. Aerodynamic particle size does not only depend on particle diameter, but also on particle shape and density, which determine how the particle will behave when moving with the air flow. Aerodynamic particle size is the diameter of a sphere with unit density having the same aerodynamic behaviour (terminal settling velocity in still air) as the observed particle and is given by the mass median aerodynamic diameter (MMAD). The MMAD is a cut-off particle size in which 50% of the mass of the aerosol is smaller and the other 50% is larger than the referred parameter [98]. As aerodynamic particle size is a result of particle dispersion in the air stream, dispersion characteristics of the formulation and the device used for dispersion are important influencing factors for the resulting MMAD. For pulmonary formulations, the number of fine particles in the inhalable range can be calculated from aerodynamic characterisations (e.g., in an impactor) and serves as an important parameter. It is given as the fine particle fraction (FPF) of either the loaded dose or the dose being emitted from the respective device. With respect to the therapeutic target of the vaccine, different regions in the lung may be interesting for delivery. Larger particles >5 µm could be used for diseases where the pathogen colonises of the upper part of the bronchi such as *Bordetella pertussis* and *Chlamydia pneumonia*. Small particles <3 µm are able to diffuse into the deep lung and may be used in the prevention of infections from *Streptococcus pneumonia* and *Bacillus anthracis* [16]. Depending on the dispersion characteristics of the formulation it may needed to be modified to the Nano-in-Micro (NiM) formulation in order to increase the dose delivered to the lung. To increase the FPF of a microparticulate powder, interactive mixtures can be used, where the fine vaccine formulation adheres to a larger carrier, with this minimising agglomeration and adhesion and easing bulk handling, and is separated during inhalation [99,100]. This leaves the large carrier in the oropharynx, whereas the smaller particles are entrained in the inhalation flow to reach the deep lung. Other approaches make use of a fine particulate excipient to increase dispersion [101]. More advanced modifications could take into account in which region the vaccine formulation should deposit and could make use of broader or smaller particle size distributions of the formulation or even monodisperse particles in order to reach the targeted area.

A vaccine formulation for the lung will be quite restricted in the choice of excipients, as the list of substances already approved for use in pulmonary dosage forms is limited. Apart from lactose, which is used as carrier in many dry powder formulations in the treatment of asthma or COPD, mannitol, glucose and sorbitol as well as some surfactants, some solvents and a limited number of polymers could be used without the need for registration of a novel excipient including all toxicity and safety tests [102,103]. Similar to a nasal vaccine NiM formulation, the matrix excipient needs to be capable for rapid dissolution in the lung mucus or the surfactant fluid in order to release the vaccine carrier [103]. If the formulation has a sufficient FPF for efficient lung delivery, it should not cause irritancy as this effect is normally caused by larger particles impacting in the oropharynx. In the delivery of high-dose antibiotics, single doses of as much as 4 × 28 mg (from the Tobi Podhaler, Novartis Pharmaceuticals) have been reported to be delivered without adverse reactions [104]. Similar to nasal formulations, pulmonary formulations do not need to be sterile, but are usually required to have a very low level of microbial contamination, which practically results in aqueous nebuliser preparations to be provided sterile.

### 9.3. Respiratory Vaccines in Research and Development

In the last centuries a lot of work and research has been done on respiratory vaccine delivery. The particulate vaccine delivery system is a growing technology and is increasingly used strategically in vaccine design. Packaging antigens in particles changes its capturing and processing by antigen presenting cells. In principle, we differentiate between four particle effects [105], which are influenced by different properties of the particle systems. First, modulation of the innate immune system, e.g., using polystyrene, PLGA or alum particle. Second, modulation of quality and quantity of antigen presentation, e.g., depot effect controlled through particle dissolution rate. Third, targeting dendritic cells, e.g., with amphiphilic polymers, or targeting cell compartments, e.g., with usage of surface charge. Fourth, enhancing uptake of antigen, e.g., with positive surface charges, in antigen presenting cells, or particle entrapment in dendritic cells [105].

Polymeric particles are the most stable particle vehicles. Different polymers are described in the literature comprising polylactide (PLA), polyglutamic acid (PGA), polylactid-co-glycolic acid (PLGA), polymethyl methacrylate (PMMA), polycaprolactone (PCL), cationic polymers, e.g., polyethyleneimine (PEI) and biopolymers, e.g., chitosan and alginate.

Various examples of polymers in mucosal vaccination as well as advantages and disadvantages of the polymeric nanoparticles [69,106,107] are described in literature. Rice-Ficht and coworkers discuss different vaccine technologies with controlled release and adjuvant effects that occur during encapsulation [105]. They speak of a single-dose pulsed release using a mixture of PLA and PLGA and sufficient immunisation through a single dose by encapsulating for example subunit vaccines with PLA and PLGA.

There are also numerous examples of nasal immunisation in the field of polymers. Jaganathan and Vyas developed surface-modified PLGA microspheres with chitosan [108]. A recombinant hepatitis B surface protein was used as an antigen. As a result, they observed a lower mucociliary clearance of the modified PLGA in comparison to unmodified PLGA and they could measure a humoral and a cellular immune response after nasal administration. Pawar and coworkers used a similar approach [109]. They also encapsulated the hepatitis surface antigen in PLGA and coated this particle with chitosan. Additional, glycol-chitosan-coated PLGA nanoparticles were prepared. Because of the nanoparticle size and a higher mucoadhesive effect of glycol chitosan, glycol-chitosan-coated PLGA nanoparticles seemed to induce a higher systemic and mucosal immune response.

Chitosan has been used as polymer for micro- and nanoparticulate preparations in many studies, as it shows numerous beneficial characteristics for mucosal administration such as mucoadhesivity and penetration-modulating properties. Ilum and coworkers explain in their paper the mechanism of mucoadhesive properties of chitosan. As a cationic polymer, it can bind to negatively charged materials. The mucus at the surface of the respiratory tract contains mucin. Sialic acid, which is a significant component of mucin, has a negative charge at physiological pH. As a result, chitosan can electrostatically interact with mucin [110].

Heidland describes in a study the particle formation method of ionic gelation [111]. Chitosan as mucoadhesive biopolymer with adjuvant activity [112] was dissolved in diluted acetic acid and carboxymethyl cellulose (CMC) or sodium deoxycholate (DOC) as a counter ion, respectively, in water. Negatively charged dissolved CMC or DOC was dripped to positively charged dissolved chitosan. Particles were formed by ionic interaction and size could be tuned both by the chitosan quality and by the ratio of chitosan and counter ion [113]. Ovalbumin was added as a model antigen to the solution with chitosan and got incorporated into the nanoparticles upon particle formation. The prepared nanoparticle suspension was then spray dried with mannitol to form Nano-in-Microparticles to allow dry-powder inhalation or dry-powder nasal administration, respectively [114]. As an alternative to the method of spray drying, freeze-drying can also be used to immobilise the nanoparticles, as described in the dissertation by Buske [115].

Similar particles being loaded with ovalbumin as model antigen have been tested with respect to their in vitro and in vivo activity with the aim to use them as therapeutic vaccine. It was shown that antigen being incorporated in chitosan-CMC nanoparticles is 10 times more effective in creating immunogenic cytokine levels and that particle uptake, antigen processing and cross-presentation can be induced in vitro in mouse and human cells [116]. In an in vivo study [117], a mild humoral and good cytotoxic immune response can be achieved after pulmonary instillation in combination with cAMP as mucosal adjuvant.

In her dissertation, Trows describes the preparation of chitosan microparticles as antigen carriers by spray drying [118]. For comparison, agarose nanoparticles were produced by nanoprecipitation. Both particulate vaccine systems have been assessed in a mouse model after nasal dry-powder administration and without the use of further adjuvants created higher local cellular immune responses than ovalbumin alone [119].

Different antigens were tested in various preclinical studies of pulmonary vaccine in mice, rats, macaques or guinea pigs. The following diseases are the focus of preclinical development: influenza, measles, hepatitis B, diphtheria [120], anthrax, *Yersinia pestis*, *Bordetella pertussis* [16], tuberculosis and Bacillus Calmette–Guerin (BCG) [121]. Several vaccines with different target groups were already tested in clinical trials: measles (+/− rubella, +/− mumps), human papilloma virus, *Streptococcus pneumonia* [120].

To increase the stability of the measles vaccine, Sievers and coworkers developed a dry-powder measles vaccine which is administered by oral inhalation or nasal–orally via a face mask [122,123,124]. The first phase I clinical trial was performed successfully (NCT01557699, 2013). As mentioned in Section 5, an aerosolised measles vaccine was developed in the 1980s. Since then, numerous children have been treated with it. As the study situation had been very inconsistent, Low and coworkers conducted a randomised, controlled trial of an aerosolised vaccine against measles involving children from 9.0 to 11.9 months of age in 2015 [125]. The data showed that the aerosolised vaccine against measles was immunogenic, but in terms of the seropositivity rate, inferior to the subcutaneous vaccine.

More recently, two nasal vaccines against seasonal influenza were approved by the authorities: Flumist/Fluenz (MedImmune/Astra Zeneca) for the US and Europe, respectively, and Nasovac (Serum Institute of India Ltd.) in India. Both vaccines make use of an attenuated influenza virus formulated in a liquid, which must be stored cold and is administered by a nasal sprayer. The vaccines have been administered to several million patients so far without any reports of severe adverse events or vaccine failure [126] and it was shown to produce a long-lasting, humoral and cellular immune response which closely resembles natural immunity. Further, nasal vaccination against influenza provides increased protection against virus drift variants and, especially, infants and children are better protected than with the inactivated, injectable influenza vaccine [96].

Audouy and coworkers used the process of spray-freeze drying to produce a dry-powder influenza vaccine for pulmonary vaccination [127]. Inulin as a cryoprotector and different types of inactive viruses of influenza were suspended in HEPES buffered saline. This dispersion was further spray-freeze dried in liquid nitrogen with a two-fluid nozzle. In in vivo mouse experiments for pulmonary immunisation with this dry powder obtained comparable protection to one single intramuscular immunisation with an injection of a subunit vaccine.

Pulmonary immunisation is of particular interest in developing countries, where the use of intramuscular vaccines is often associated with problems. Tuberculosis and hepatitis B are two of the world’s leading infectious diseases and the treatment and prevention of these diseases are currently important subjects of research.

Formulation of tuberculosis vaccines as dry powder with nasal or pulmonary administration may provide attractive options. Källenius and coworkers [128] discuss in their paper if new tuberculosis vaccines should be administered intranasally. They consider protection against different types of mycobacteria while using live BCG, killed BCG with adjuvant subcomponent or recombinant adenovirus-based vaccines for intranasal vaccination. However, they also mentioned some disadvantages for nasal application, e.g., the potential access to the central nervous system and in connection therewith, the composition of the formulation. This point is also an aspect in the paper of Fourie and coworkers [129]. They developed a nasal dosage form for pulmonary administration as a preferential route for deep-lung deposition of dry powder tuberculosis vaccines and talking about spray-dried formulations with leucine, PLGA, sucrose and trehalose. Garcia-Contreras and coworkers [130] also used a relatively high amount of leucine (95%) for the spray-drying process with the mycobacteria (5%). After nine months in a refrigerator (4 °C) almost no negative effects of biological activity and other characteristics in comparison to day 1 were detectable. In in vivo experiments with guinea pigs, pulmonary application had a higher reduction of viable bacteria per millilitre of tissue homogenate than parenteral immunisation, compared with untreated controls.

Dry powder formulations of hepatitis B could also be candidates for pulmonary administration. For this purpose, Muttil and coworkers [131] created nanoparticle-aggregate formulations, made of PLGA (core) and PEG (shell) with recombinant hepatitis B surface antigen by the double emulsion method. The aqueous nanosuspension (10%) was then spray dried with leucine (90%). In in vivo experiments with guinea pigs, the formulation showed a protective antibody level and high local IgA antibody titres after pulmonary administration, which was higher compared to intramuscular administration.

These examples show that pulmonary immunisation with dry-powder formulations are possible and would be particularly useful for pulmonary diseases.

## 10. Pulmonary and Nasal Administration Devices

### 10.1. Nasal Administration

For nasal administration and deposition of vaccine formulations, properties of the devices, in addition to the formulation properties, have a great effect, especially with respect to dispersion capacity and spray velocity. Usually, nasal application requires a droplet/particle size above 10 µm. Devices that can be used for nasal administration can be found in Table 5.

#### 10.1.1. Liquid Preparations

Liquid nasal formulations are mainly aqueous solutions. Drops delivered with a pipette are the oldest forms of nasal delivery and often used in infants. Liquid has to be sucked into a glass pipette and then dropped into the nostrils. The handling of nose drops for a perfect distribution in the nose is complicated, which leads to the fact that the popularity and also the compliance are rather low.

Squeeze bottles, where a bottle need to be squeezed for atomising the drug through a jet outlet, metered-dose spray pumps and single-dose spray devices are possibilities for mechanical spray pumps. Metered-dose spray pumps are the most used ones at the market. They offer high reproducibility of the emitted dose and it is possible to avoid preservatives because of special mechanisms or constructions, e.g., aseptic air filters. Most over-the-counter (OTC) drugs, like decongestants are available in this device. For drugs with a narrow therapeutic window, expensive drugs or vaccines, single-dose spray devices are preferred. For this purpose, a conical nosepiece with a spray tip can be connected to a normal syringe. This device is, for example, used to deliver FluMist^®^, the influenza vaccine.

For single-dose or bi-dose applications, simple systems are available, in which a liquid container is pierced and emptied upon actuation (e.g., the Unitdose System from Aptar, France, or the Unidose Xtra from Bespak, UK).

Propellant-driven spray systems, e.g., nasal pressurised metered-dose inhalers, produce, like spray pumps, a localised deposition, but because of the quick evaporation of hydrofluoroalkanes (HFA) a noticeable “drip-out” could be less problematic.

Electrically powered nebulisers break up solutions or suspensions into small droplets with pulsation of a membrane or a vibrating mesh. They can be directly inhaled into the mouth or the nose. Because of the special mechanism characteristics and in connection therewith the deposition can be modified and used in a more targeted manner. Usually, nebulisers are bigger, not handheld devices [132].

#### 10.1.2. Dry Powder Devices

Among other things, particle size as a result of the formulation and the dispersion capacity of the device has an effect on nasal deposition and absorption. The function of a nasal powder device is based on one of three principles: a compressible compartment to provide a pressure and create a plume, breath-actuated inhalers and nasal insufflators, where a mouthpiece is connected with a nosepiece and the patient blows the powder into its own nose while exhaling into the mouthpiece.

Different nasal powder inhalers already exist and have to be actuated by breathing. Astra Zeneca designed a modified Turbohaler for nasal administration (Rhinocort Turbohaler^®^, Astra Zeneca, Södertälje, Sweden). This multi-dose inhaler device is marketed for nasal rhinitis.

Nasal powder sprayers have the advantage in comparison to nasal powder inhalers that the patient does not have to apply the powder with his own inspiration. Fit-lizer^TM^ (SNBL Pharma, Japan) is a capsule based, single-dose powder device. The top and the bottom of a capsule are cut off by sharp blades and the compressed plastic chamber passes the air through the device and the powder is emitted.

The unit dose powder device (UDS powder, Aptar, France) is a single-dose, single-use device in which an air-filled compartment is compressed, a pin ruptures a membrane and the released pressure emits the plume of powder [132].

Nasal powder insufflators are breath-powered technologies. OptiNose (Optinose UK Ltd., Swindon, UK) developed this breath-powered, bi-directional nasal delivery technology. The patient utilises the exhaled breath to deliver the drug to the nose. Because of a special mechanism within the administration process drug deposition, clearance patterns and clinical device performance are unique and promising. OptiNose can be used for both liquid and dry powder formulation and vaccines have already been tested with the device [132].

### 10.2. Pulmonary Administration

For pulmonary administration of vaccine formulations, device dispersion must result in an inhalable fine aerosol with aerodynamic characteristics suitable for lung delivery, which normally requires an aerodynamic particle size below 5 µm. Devices that can be used for pulmonary administration can be found in Table 6.

#### 10.2.1. Liquid Preparations

For liquid preparations, nebulisation is a feasible approach. Here, devices like mesh nebulisers, ultrasonic nebulisers or air jet nebulisers such as the Mexican device, may be utilised. Pulmonary nebulisers are normally designed to produce a fine aerosol which can penetrate deep into the lung. Unfortunately, they sometimes have been reported to adversely affect macromolecules such as antigenic proteins due to the energy stress of the nebulisation procedure. This can result in a loss of potency of the vaccine formulation [16]. Similar to nasal administration, liquid solution or suspension preparations can also be administered by a pMDI, but the pharmaceutically available propellants are generally not considered as suitable dispersants for biologicals such as vaccine preparations [16].

#### 10.2.2. Dry Powder Inhalers

The most promising pulmonary vaccine delivery devices are dry powder inhalers. Sievers and coworkers used two types of dry powder inhalers, which actively disperse the measles vaccine powder and are meant for either oral inhalation or inhalation with a face mask from a reservoir, where the dispersed powder cloud is captured. Actuation is performed by actively generating an air puff either by a squeeze bulb or by an air-filled syringe [123]. The air puff entrains the powder, which is dispersed in a powder reservoir, from where it is subsequently inhaled. During storage, the vaccine powder is either stored in single-dose blisters in the case of PuffHaler or in special “capsules” having thin plastic films on either side, which rupture upon actuation [133]. Another possibility is to store the single vaccine dose in gelatine or HPMC capsules, which can then be used in the commercially available Cyclohaler device or other capsule-based dry powder inhalers such as the Unihaler [134] or the Twister (Aptar Pharma, France). The principle of operation is the same for all of these passive dry-powder inhalers. The capsule is placed in the device, it is pierced or cut open and upon patient inhalation, the powder is entrained in the inhalation air flow and gets inhaled to the lung. The powder is dispersed passively by the patient’s inhalation. Hence, efficiency depends on the patient’s inhalation capabilities and manoeuvre. For vaccination applications, single or dual use systems might be of advantage as vaccination should best be carried out as a single shot, perhaps with one or two boosts after certain time intervals. Here, a dual dose system such as the TwinCaps inhaler (Hovione, Portugal) could be used. Two doses are pre-filled in capsules within the device and can be inhaled subsequently. The inhaler itself is disposable after use. Other single-dose devices made for disposal after use are, amongst others, the Twincer (Stichting Groningen Center for Drug Research, The Netherlands) and the DryPod (now resQhaler, Aespira, Israel) [135]. Both inhalers have originally been developed for dry-powder inhalation of high-dose drug products, but they could also be used for vaccination. In the resQhaler, the powder dose is prefilled in a mesh-like reservoir, which starts beating upon patient inhalation through the device and with this release, the powder (ActiveMesh technology). In the Twincer device [136], the powder is sealed in a blister, which is opened directly prior to inhalation. The powder is dispersed with the help of two classifier chambers and is entrained in the patient’s airflow upon inhalation.

For all types of inhalers, it is important that the vaccine formulation is compatible with the material it directly comes into contact with. This might be an issue for formulations of protein antigens being filled in gelatine capsules, but also for inhaler materials, which could adsorb formulation components by electrostatic interactions with this minimising the emitted dose or could lead to forced degradation of the antigen. In general, the device plays an important role for the dispersion and, hence, delivery of the formulation. Therefore, a vaccine formulation for pulmonary delivery must always be developed and characterised together with the respective inhalation device.

## 11. Conclusions

Vaccination via the respiratory tract is an attractive strategy for a number of applications in preventive and therapeutic immunisation. The mucosa-associated lymphoid tissue is an excellent induction site as both systemic and local immune response along with a high cytotoxic effect and a cellular immune response can be used. The multitude of formulation possibilities and the comparatively simple handling of the devices leave room for the development of new ideas. Precisely for these reasons, the respiratory tract is an application route that will be increasingly used for vaccination in the future.

## 12. Patents

Hanefeld, A.; Weigandt, M.; Wolf, M.; Knolle, P.; Schröder, M.; Scherließ, R.; Walden, P.; Diedrich, A.; Steckel, H.; Baleeiro, R.B. Antigen-loaded chitosan nanoparticles for immunotherapy. Merck Patent Gesellschaft (2014), WO2015/185180A1.

## Figures and Tables

**Table 1:** Various alternatives to intramuscular application [5].

Cutaneous Immunisation	Mucosal Immunisation
Epidermal powder immunisation	Ocular immunisation
	Nasal immunisation
Liquid-jet immunisation	Pulmonary immunisation
	Oral immunisation
Topical application	Vaginal immunisation
	Rectal immunisation

**Table 2:** Location of immune cells within the respiratory tract [6,8,9,10,11,12,13].

Parts of the Immune System	Location	Cells/Structures
Epithelial compartments with immunocompetent cells	Nose/lung	Macrophages
		Dendritic cells (DCs)
		M cells
		T and B lymphocytes
Lymphoid structures of the nose and the bronchus	Nose: nasopharynx and tonsils	Nose-associated lymphoid tissue (NALT)
	Larynx	Larynx-associated lymphoid tissue (LALT)
	Lung: upper and lower airways (branching site)	Bronchus-associated lymphoid tissue (BALT)
Lymph nodes		T and B cells

**Table 3:** Stabilisers used in vaccines during the drying process [54,56,57].

Vaccine	Method	Stabiliser
Influenza	Spray drying	DPPC/HES (dipalmitoyl phosphatidylcholine/hydroxyethyl starch)
		Inulin [59]
	Spray-freeze drying	Arginine
		Dextran
		Lactose
		Mannitol
		Trehalose
	Freeze drying	Dextran
		HYAFF (esterified hyaluronic acid) microspheres
		Inulin
		Sorbitol
		Trehalose
	Air drying	d-xylose
Smallpox	Freeze drying	Mannitol
Measles, mumps and rubella	Freeze drying	Sorbitol
		Sucrose
Hepatitis B	Spray-freeze drying	Inulin
		Dextran
		Trehalose

**Table 4:** Mucosal adjuvants in the respiratory tract [69,70].

Mucosal Adjuvant	Mechanism	Immune Response in the Respiratory Tract
Lipopolysaccharide (LPS)–protein complexes (endotoxins): - Cholera enterotoxin (CT) - Heat-labile enterotoxin (LT) from *E. coli*	Enhance antigen-specific mucosal IgA and systemic IgG responses to administered proteins [71]	Yes [16]
Monophosphoryl lipid A (MPL)	Activate cells via Toll-like Receptor 4 (TLR4) [72]	Yes [73]
Muramyl dipeptide (MDP)	Enhance the cell-mediated immune response [74]	Yes [75]
Oligonucleotids (CpG)	Stimulate B cells to proliferate and secrete immunoglobulins, activate APCs and stimulate cytokine production [76]	Yes [77]
Saponins like QuilA (e.g., ISCOMS)	Improve T cell responses and antigen uptake by APC [78,79]	Yes [79]
Non-ionic block polymers (Poloxamers)	Enhance antigen presentation by binding protein antigens to the surface of the oil droplets [80]	Yes [76]
Dehydroepiandrosterone (DHEA)	Increase cell-mediated immunity [80]	Yes [81]
Cytokines - Il-1 - Il-12	Enhance B cell growth (Il-12) and influence the differentiation of Th cells (Il-1) [82]	Yes [82]

**Table 5:** Examples of devices for nasal administration.

Devices for Liquid Preparations		Dry Powder Devices	
Device	Company	Device	Company
Nasal pressurised metered-dose inhaler	3M, USA	Unit dose powder device	Aptar, France
Unitdose System	Aptar, France	Turbohaler	Astra Zeneca, Sweden
Unidose Xtra	Bespak, UK	OptiNose	Optinose UK Ltd.
Nebuliser, e.g., ViaNase	Kurve Technology, USA	Fit-lizer	SNBL Pharma, Japan
OptiNose	Optinose UK Ltd.		
Single-dose spray devices - Syringe with conical nosepiece with a spray tip, e.g., MAD Nasal	Teleflex, USA		
Metered-dose spray pumps			

**Table 6:** Examples of devices for pulmonary administration [16,128,133,134,135,136].

Devices for Liquid Preparations		Dry Powder Inhalers	
Device	Company	Device	Company
Pressurised metered-dose inhaler	Aptar, France 3M, USA Bespak, UK	ResQhaler	Aespira, Israel
Nebuliser		Twister	Aptar, France
- Mesh nebuliser, e.g., I-neb Adaptive Aerosol Delivery System	Philips N.V., The Netherlands	TwinCaps	Hovione, Portugal
		Twincer	Stichting Groningen Centre for Drug Research, The Netherlands
- Ultrasonic nebuliser, e.g., aerosonic	Flores Medical GmbH, Germany	Cyclohaler	
		PuffHaler	
- Air jet nebuliser, e.g., PariLC	Pari GmbH Germany	Unihaler	
